# Mitigation of mycotoxin residues and activation of endogenous stem cells in broiler chickens using a toxin binder: Implications for meat safety and performance enhancement

**DOI:** 10.14202/vetworld.2025.1850-1862

**Published:** 2025-07-08

**Authors:** Erma Safitri, Hery Purnobasuki, Tita Damayanti Lestari, Suzanita Utama, Rimayanti Rimayanti, Mirni Lamid, Mutmainah Wardatul Jannah, Siti Darodjah, Goo Jang, Mitsuhiro Takagi

**Affiliations:** 1 Department of Veterinary Science, Division of Veterinary Reproduction, Faculty of Veterinary Medicine, Universitas Airlangga, Surabaya, Indonesia; 2 Department of Biology, Faculty of Science and Technology, Universitas Airlangga, Surabaya, Indonesia; 3 Department of Veterinary Science, Division of Animal Husbandry, Faculty of Veterinary Medicine, Universitas Airlangga, Surabaya, Indonesia; 4 Department of Veterinary Science, Division of Veterinary Reproduction Undergraduate Student of Veterinary Medicine Faculty, Universitas Airlangga, Surabaya, Indonesia; 5 Department of Animal Production, Animal Husbandry Faculty, Universitas Padjadjaran, West Java Indonesia; 6 Department of Theriogenology, College of Veterinary Medicine, Seoul National University, Seoul, Republic of Korea; 7 Laboratory of Theriogenology Joint Faculty of Veterinary Medicine, Yamaguchi University, Yamaguchi, Japan

**Keywords:** aflatoxin, broiler chicken, feed conversion, health, mycotoxin residue, ochratoxin, performance index, stem cells, toxin binder

## Abstract

**Background and Aim:**

Mycotoxin contamination in poultry feed, particularly with aflatoxin B1 (AFB1) and ochratoxin A (OTA), poses significant threats to broiler health, meat quality, and consumer safety. Toxin binders are commonly used to mitigate these effects; however, their impact on endogenous stem cell activity and overall broiler performance remains underexplored. This study aimed to evaluate the efficacy of a commercial toxin binder in reducing AFB1 and OTA residues in broiler meat, inducing endogenous stem cell production, and improving growth and feed performance indices.

**Materials and Methods:**

Twenty Cobb broilers were randomly assigned to four groups: Negative control (C−), positive control with mycotoxin-contaminated feed (C+), treatment 1 (T1: 1.1 g/kg binder), and treatment 2 (T2: 1.6 g/kg binder). Broilers were fed for 35 days. AFB1 and OTA levels in pectoral muscles were quantified using high-performance liquid chromatography, while endogenous stem cell markers (CD34+, CD45+, CD105−) in spleen tissue were assessed through flow cytometry. Growth parameters, feed conversion ratio (FCR), and performance index were also evaluated.

**Results:**

AFB1 and OTA residues were significantly reduced in T1 and T2 compared to C+ (p < 0.05), with T2 showing the lowest levels (0.0023 μg/mL and 0.073 μg/mL, respectively). Flow cytometry revealed that T2 significantly induced endogenous stem cells (35.62% ± 2.16) compared to all other groups. The highest average daily growth occurred in T1 (68.78 ± 4.78 g/day), while the best FCR (1.38 ± 0.079) and performance index (386.2 ± 14.34) were also recorded in T1. No mortality occurred in any group.

**Conclusion:**

Administering a toxin binder at 1.6 g/kg effectively reduced AFB1 and OTA residues and significantly activated endogenous stem cells, suggesting a protective and regenerative effect. Meanwhile, a dose of 1.1 g/kg yielded optimal growth performance and feed efficiency. These findings support the dual functional role of toxin binders in enhancing broiler meat safety and physiological resilience.

## INTRODUCTION

Feed quality is a pivotal determinant of livestock productivity, particularly in broiler production systems. Optimal feed must be nutritionally balanced, providing adequate levels of protein, energy, vitamins, and minerals to support rapid growth and health maintenance in poultry species [1]. However, the integrity of feed is highly susceptible to degradation under improper storage conditions, which foster fungal proliferation and subsequent mycotoxin contamination. These toxic secondary metabolites, particularly when present as mixed mycotoxins, originate from various toxigenic mold genera and can significantly compromise both feed safety and animal health [2]. Mixed mycotoxins exert multifaceted deleterious effects in poultry, including diminished growth performance, impaired reproductive function, suppressed immunity, and reduced availability of endogenous stem cells necessary for tissue repair [3]. Clinical manifestations such as decreased feed intake and poor body weight gain are frequently observed. Moreover, the consumption of contaminated feed leads to the bioaccumulation of mycotoxins in edible tissues and organs, thus posing a consequential risk to public health through the food chain.

To mitigate these adverse outcomes, the application of mycotoxin-binding agents, commonly referred to as toxin binders, has emerged as a critical intervention strategy. When incorporated into poultry feed, toxin binders adsorb mycotoxins within the gastrointestinal tract, thereby preventing their systemic absorption and minimizing residue accumulation in meat and other animal products [3, 4]. A comprehensive evaluation of broiler performance necessitates the use of metrics such as the performance index, which integrates key parameters: Slaughter-ready body weight, feed conversion ratio (FCR), harvest age, and survival rate during the rearing period [1]. The FCR, calculated as the ratio of feed consumed to body weight gained, serves as a cornerstone for assessing feed efficiency. Lower FCR values indicate superior conversion efficiency, which is often reflected in a higher performance index [5]. Given the multifactorial impact of mycotoxins, research is warranted to assess the efficacy of toxin binders in reducing aflatoxin B1 (AFB1) and ochratoxin A (OTA) residues in broiler meat. High-performance liquid chromatography (HPLC) provides a precise method for quantifying residues. In parallel, the role of toxin binders in stimulating endogenous stem cell activity, assessed through flow cytometry, provides insight into regenerative responses that enhance tissue repair and overall meat quality. Endogenous stem cells, being multipotent, play a central role in restoring tissue homeostasis following damage induced by toxic insult [6–8]. These cells are mobilized and guided through complex interactions with the immune system, extracellular matrix, and regulatory growth factors to facilitate regeneration. Thus, improvements in meat quality may also be indirectly evaluated through enhanced biological repair mechanisms in addition to conventional performance indices. Despite the widespread incorporation of mycotoxin binders in poultry production systems, the current literature lacks comprehensive studies that simultaneously assess their dual function: (1) reducing mixed mycotoxin residues in edible tissues and (2) enhancing endogenous tissue repair mechanisms through stem cell mobilization. Most existing research has focused primarily on growth performance, immune response, or residue quantification individually, without integrating these physiological and toxicological endpoints into a unified experimental framework. Moreover, while AFB1 and OTA are among the most prevalent and hazardous mycotoxins affecting broiler health, limited studies have evaluated their combined toxicity under controlled exposure and the specific capacity of binders to mitigate this risk. Crucially, the regenerative potential of toxin binders – particularly their influence on the mobilization of endogenous hematopoietic stem cells (HSCs) – remains largely unexplored *in vivo*. This constitutes a significant gap in understanding the broader physiological implications of binder supplementation beyond toxin sequestration. Therefore, there is a critical need for an integrated study that evaluates the safety, regenerative stimulation, and productive outcomes of broilers exposed to mixed mycotoxins and treated with commercial toxin binders.

The present study aimed to evaluate the efficacy of a commercial mycotoxin binder in mitigating the toxic effects of a mixed mycotoxin challenge (AFB1 and OTA) in broiler chickens. Specifically, the objectives were threefold (1) to quantify the residual concentrations of AFB1 and OTA in broiler meat using HPLC following dietary administration of the toxin binder, (2) to assess the mobilization of endogenous HSCs (CD34+, CD45+, CD105−) in spleen tissues through flow cytometry, as an indicator of systemic regenerative activity, and (3) to examine the effects of binder supplementation on zootechnical parameters, including body weight gain, FCR, performance index, and survival rate. By integrating toxicological, regenerative, and performance-based endpoints, this study seeks to elucidate the comprehensive benefits of toxin binder supplementation and its implications for food safety, animal welfare, and poultry production efficiency.

## MATERIALS AND METHODS

### Ethical approval

All animal handling procedures were conducted in compliance with ethical standards. The experimental protocol was reviewed and approved by the Animal Care and Use Committee of the Faculty of Veterinary Medicine, Universitas Airlangga (Approval No. 1.KEH.033.02.2023). Humane treatment and welfare of broilers were ensured throughout the study duration.

### Study period and location

The study was conducted from July to September 2023 at the experimental animal cage of the Faculty of Veterinary Medicine, Universitas Airlangga, Surabaya.

### Experimental design

A total of 20 Cobb strain broiler chicks (final stock) were randomly assigned to four experimental groups (n = 5/group). The chicks were reared under standard broiler management conditions until slaughter age (35 days). The study employed a true experimental, post-test-only control group design to evaluate the impact of toxin binder supplementation on (1) endogenous stem cell biomarkers, (2) residual levels of AFB1 and OTA in broiler meat, (3) average daily weight gain, and (4) feed conversion and performance index parameters. A completely randomized design was implemented to ensure data reliability and minimize experimental bias.

### Animal management and housing conditions

One week before chick arrival, the broiler house, cages, and all associated equipment were disinfected using standard protocols. Day-old chicks (DOCs) were maintained under standardized environmental conditions, including appropriate ventilation, temperature, and humidity. Birds were fed *ad libitum* a commercial starter feed (Hipprovite CP511, PT. Charoen Pokphand Indonesia), formulated according to National Research Council recommendations. Clean drinking water was always available.

### Mycotoxin contamination and feed preparation

To simulate real-world dietary exposure to mycotoxins, feed was artificially contaminated using high-purity laboratory-grade standards of AFB1 (product code A-1100, Fermentek Ltd., Israel) and OTA (product code GC40762, Glpbio, USA). Each toxin was added at a concentration of 0.16 mg/kg of feed. The compounds were dissolved in methanol or ethanol for uniform dispersion and thoroughly mixed into the basal diet using a mechanical mixer. The feed was air-dried to evaporate residual solvent and stored in sealed, moisture-free containers until it was used.

### Toxin binder administration

The 20 broiler chickens were divided into four treatment groups:

- Negative control (C−): Basal feed without mycotoxin contamination or binder
- Positive control (C+): Feed contaminated with AFB1 and OTA without binder
- Treatment 1 (T1): Contaminated feed + 1.1 g/kg toxin binder
- Treatment 2 (T2): Contaminated feed + 1.6 g/kg toxin binder.

The toxin binder used was Mycofix Plus (Biomin GmbH, Austria), a commercial product comprising dioctahedral montmorillonite, enzymatic biotransforming agents (e.g., *Trichosporon mycoto-xinivorans*, BBSH 797), and phytogenic components. Treatments were administered from day 8 until day 35.

### Sample collection

At 35 days of age, birds were humanely slaughtered, and samples were collected for analysis. Pectoral muscle tissue was collected to evaluate AFB1 and OTA residues through HPLC. Spleen samples were harvested to assess endogenous stem cell populations using flow cytometry.

### Mycotoxin residue analysis through HPLC

Approximately 5 g of homogenized pectoral muscle tissue was extracted with McIlvaine-ethylenediaminetetraacetic acid buffer and centrifuged at 1,250 × *g* for 10 min. The supernatant was filtered for HPLC analysis.

- Instrumentation: HPLC was conducted using a C18 reverse-phase column (specifications to be provided), with a mobile phase of methanol: water (1:1) at a flow rate of 0.6 mL/min
- Detection: Samples were injected at 10 μL volumes using a calibrated syringe
- Validation parameters: Limit of detection (LOD) and limit of quantification (LOQ) were 0.0014 μg/mL and 0.0048 μg/mL, respectively. AFB1 and OTA exhibited clear, non-overlapping peaks at retention times of 16–17 min and 3.5–3.9 min, respectively. Pressure readings were closely monitored to ensure instrument safety.

### Stem cell analysis through flow cytometry

Spleens were aseptically removed and homogenized in phosphate-buffered saline (pH 7.0). The resulting cell suspensions were filtered and centri-fuged at 1,100 × *g* for 5 min at 10°C. Pellets were resuspended and divided into aliquots.

- Extracellular staining: Cell pellets were incubated with 50 µL of CD34, CD45, and CD105 polyclonal antibodies (Bioss Inc., USA) for 20 min in the dark at 20°C
- Intracellular staining: Fixation and permeabilization were performed using BioLegend reagents before secondary antibody incubation
- Instrumentation: Flow cytometric analysis was conducted on a BD FACSCalibur system (BD Biosciences, USA) with daily calibration and appropriate gating for CD34+, CD45+, and CD105− populations.

### Growth performance evaluation

Body weights were recorded at 7, 21, and 35 days. The FCR was calculated as the ratio of feed consumed to weight gained. Survival rate and performance index were calculated using standard industry formulas, incorporating final body weight, feed intake, age at harvest, and mortality data.

### Statistical analysis

All quantitative data were analyzed using the Statistical Package for the Social Sciences version 25.0 (IBM Corp., NY, USA). A one-way analysis of variance was employed to compare treatment groups, followed by Duncan’s multiple range test for *post hoc* analysis. Statistical significance was accepted at p < 0.05, and data are presented as mean ± standard deviation.

## RESULTS

### HPLC analysis of mycotoxin residues

#### Sample collection and preparation

Meat samples were obtained from Cobb strain broilers at slaughter age (35 days), reared under controlled conditions at the Faculty of Veterinary Medicine, Universitas Airlangga. Pectoral muscle tissues were aseptically collected post-mortem and prepared for HPLC analysis to determine the residual levels of AFB1 and OTA.

#### AFB1 quantification and calibration

Sample analysis was conducted following the preparation of standard solutions of AFB1 at a concentration of 0.05 μg/mL. Analytical parameters such as chromatogram peak area and height were recorded and compared against calibration curves constructed from known concentrations. The regres-sion analysis demonstrated excellent linearity with an R^2^ value > 0.99. The standard curve data for AFB1 included:

- 6.78333 (0.0937 μg/mL)
- 6.58598 (0.0468 μg/mL)
- 6.49141 (0.0234 μg/mL)

**Figure 1:**
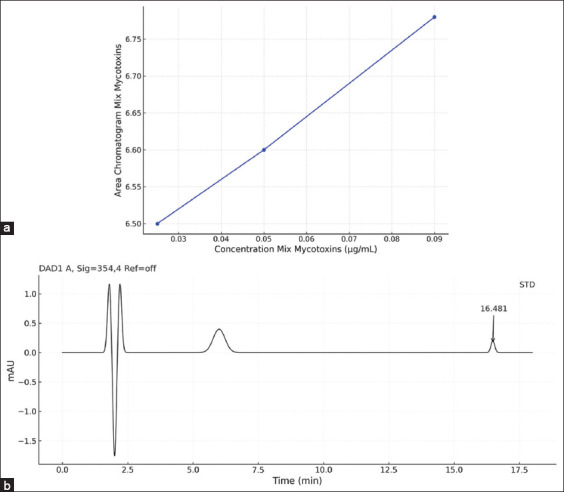
(a). Aflatoxin B1 (AFB1) calibration curve in matrix plasma (R² = 0.99) and (b). the lowest concentration of AFB1 (0.05 μg/mL) in the mobile phase.

**Figure 2:**
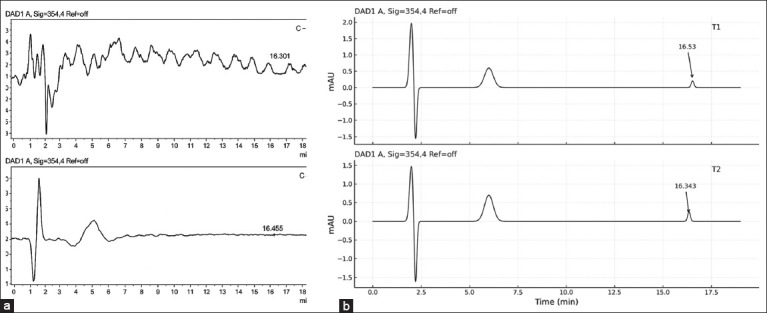
(a) HPLC chromatogram data during treatment of AFB1 in broiler chicken meat and (b) HPLC chromatogram data during treatment of OTA in broiler chicken meat. C(−): Chickens fed with commercial feed; C(+): Chickens fed with a mycotoxin-contaminated feed mixture; T1, T2: Chickens fed with a mycotoxin mixture feed at a dose of aflatoxin and ochratoxin levels were 0.1 mg/kg feed, with mycotoxin T1 dosed at 1.1 g/kg feed and T2 dosed at 1.6 g/kg feed for 8-35 days, with five repetitions. HPLC=High-performance liquid chromatography.

The resulting linear regression equation was y = 4.1573x + 6.393. The validated detection parameters included a LOD of 0.0014 μg/mL and a LOQ of 0.0048 μg/mL, with a consistent retention time of 16–17 min. Figures 1 and 2 display the calibration data and chromatogram, respectively.

AFB1 analysis was performed in triplicate to ensure accuracy. Residual concentrations in meat were calculated by averaging the peak areas observed at the 16–17 min retention window and applying the dilution factor.

### OTA quantification

#### Calibration and standard curve

**Figure 3:**
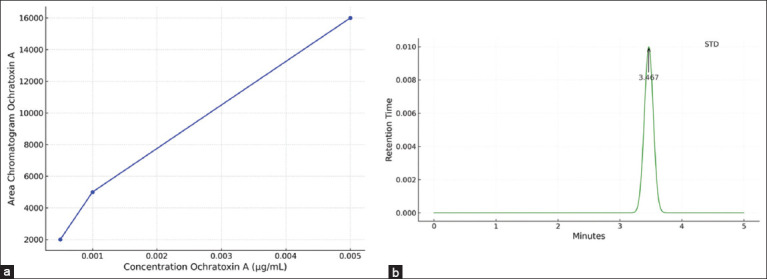
(a) The calibration curve of ochratoxin A (OTA) in matrix plasma (R² = 0.99) and (b) the lowest concentration of OTA (0.05 μg/mL) in the mobile phase.

Figure 3 presents the standard curve for OTA, which exhibited a retention time between 3.5 and 3.9 min. The calibration data are as follows:

- 16,311 (0.005 μg/mL)
- 5,132 (0.001 μg/mL)
- 2,170 (0.0001 μg/mL)

The linear regression equation derived for OTA was y = 3.06x + 2059.3, with LOD and LOQ values of 0.00029355 ppm and 0.00097852 ppm, respectively.

### Residue detection in broiler meat

OTA was undetectable in the negative control group (C−), whereas detectable levels were present in all other groups:

- C+ group: 0.730 ppm
- T1 group: 0.116 ppm
- T2 group: 0.073 ppm

**Figure 4:**
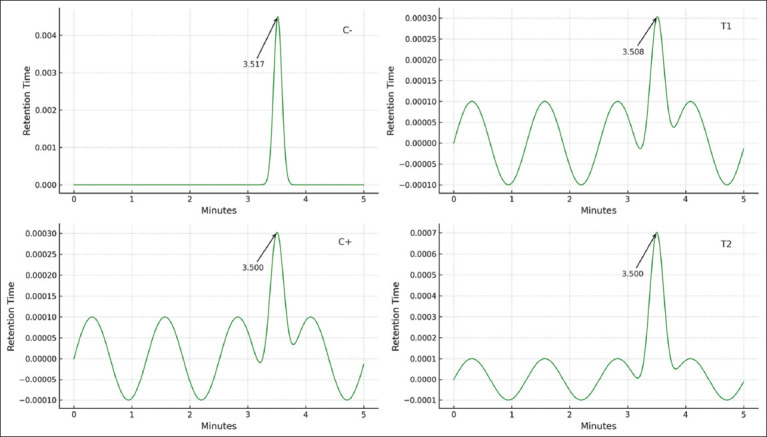
HPLC chromatogram data during treatment of ochratoxin A in broiler chicken meat. C(−): Chickens fed with commercial feed; C(+): Chickens fed with a mycotoxin-contaminated feed mixture; T1, T2: Chickens fed with a mycotoxin mixture feed at a dose of aflatoxin and ochratoxin were present at 0.1 mg/kg feed, with mycotoxin T1 administered at a dose of 1.1 g/kg feed and T2 at a dose of 1.6 g/kg feed, over a period of 8–35 days, with 5 repetitions. HPLC=High-performance liquid chromatography.

These results are summarized in Table 1, and representative chromatograms are shown in Figure 4.

**Table 1:** Intraday precision of ochratoxin A dissolution in the mobile phase.

Sample	Area chromatogram	Std concentration (µg/mL)	Concentration (µg/mL)
C−	*nd	*nd	*nd
C+	4186.3	0.073	0.730
T1	667.3	0.073	0.116
T2	423.6	0.073	0.073

* nd=not detected

#### Induction of endogenous stem cells

**Figure 5:**
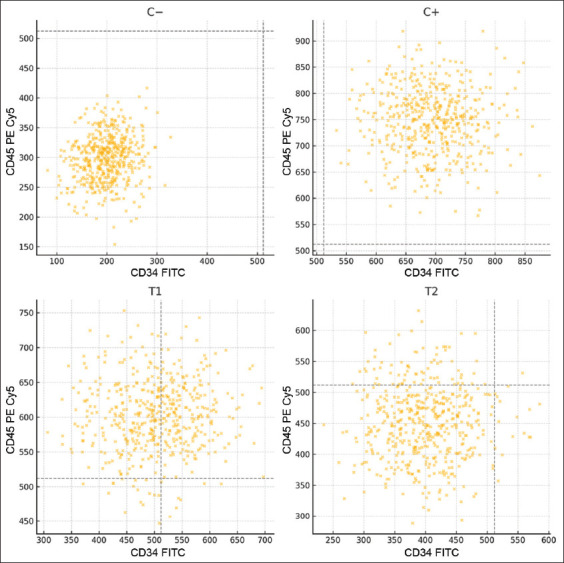
Flow cytometry results of CD34+ and CD45+ spleens from final broiler stock. Furthermore, efforts to improve meat quality can be made by observing the Performance Index, which consists of body weight ready for slaughter, feed conversion ratio, harvest age, and percentage of living chickens during the rearing period.

Endogenous HSC activity was assessed through flow cytometry by analyzing spleen tissues collected at slaughter. Cells were stained for CD34, CD45, and CD105 markers. The results revealed a significant increase in HSC mobilization in the T2 group (1.6 g/kg toxin binder) compared to the control and other treatment groups. Flow cytometric data and mean percentages of HSC markers are presented in Table 2 and visualized in Figure 5.

**Table 2:** Mean HSCs (CD34+, CD45+and CD105−) with flow cytometry in final stock.

Treatment	Mean (%) ± Standard deviation
C−	2.07^a^ ± 0.48
C+	6.48^b^ ± 0.22
T1	7.39^b^ ± 0.91
T2	35.62^c^ ± 2.16

Different letter notations in the same column indicate significant differences (p < 0.05). HSCs=Hematopoietic stem cells

### Growth performance evaluation

#### Average daily growth (ADG)

**Figure 6:**
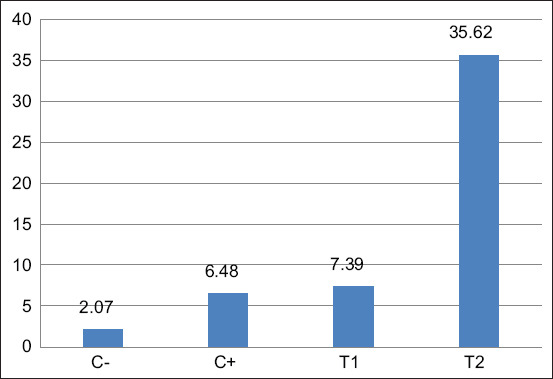
Mean of CD34+, CD45+, CD105− with flow cytometry.

Growth performance was evaluated by calculating the average daily weight gain (g/head/day) from body weight measurements taken on days 7, 21, and 35. The mean and standard deviation for each group are provided in Table 3 and illustrated in Figure 6.

**Table 3:** Mean and SD of daily body weight gain, in broilers given mycotoxin binder and exposed to mycotoxin.

Treatment	Increase in body weight daily ± SD (g/head/day)
C(−)	64.65^b^ ± 4.25
C(+)	57.50^a^ ± 2.41
T1	68.78^b^ ± 4.78
T2	67.17^b^ ± 3.63

Different superscripts in the same column indicate significant differences (p < 0.05). SD=Standard deviation

#### FCR and performance index

Feed efficiency was measured using the FCR, and productivity was assessed through the performance index. Both parameters were calculated and compared across groups.

**Figure 7:**
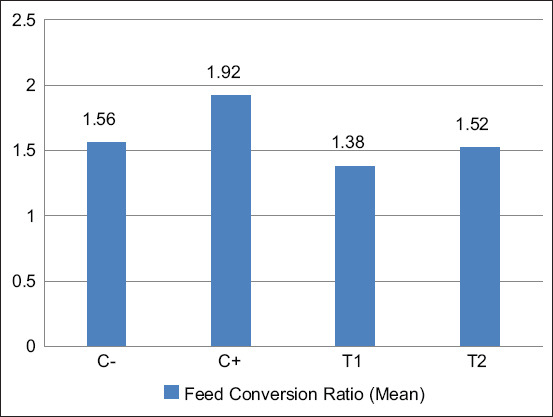
Bar histogram of the average feed conversion ratio for broiler chickens during treatment; C(−): Chickens are given commercial feed only; C(+): Chickens given contaminated feed exposed to mycotoxin mix; T1, T2: Chickens were exposed to a mycotoxin mix with aflatoxin dose of 0.1 mg/kg feed + ochratoxin 0.1 mg/kg feed and mycotoxin binder with a dose for T1 of 1.1 g/kg feed while for T2 with a dose of 1.6 g/kg feed; Treatment for 8-35 days; Repetition = 5 times.

**Figure 8:**
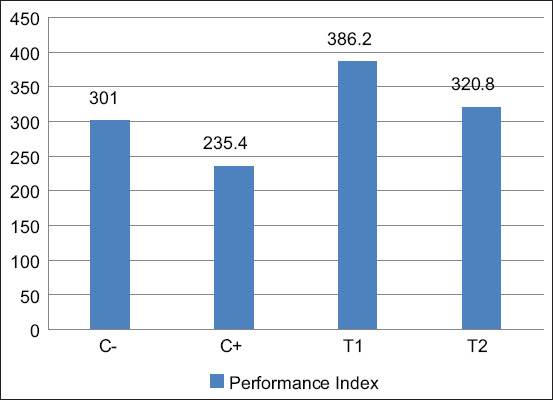
Histogram of average bar performance index for broiler chickens during treatment; C(−): Chickens are given commercial feed only; C(+): Chickens are given contaminated feed exposed to mycotoxin mix; T1, T2: Chickens were exposed to a mycotoxin mix with aflatoxin dose of 0.1 mg/kg feed + ochratoxin 0.1 mg/kg feed and mycotoxin binder with a dose for T1 of 1.1 g/kg feed while for T2 with a dose of 1.6 g/kg feed; Treatment for 8–35 days; Repetition = 5 times.

- The FCR and performance index data are summarized in Table 4
- Figure 7 illustrates the mean FCR values
- Figure 8 displays the corresponding performance index results.

**Table 4:** FCR and performance index (Mean ± Standard deviation) of broiler chickens after the administration of mycotoxin mix and mycotoxin binder.

Treatment	FCR (Mean ± Standard deviation)	Performance index ± Standard deviation
C(−)	1.56^b^ ± 0.089	301^b^ ± 30.23
C(+)	1.92^c^ ± 0.083	235.4^a^ ± 17.27
T1	1.38^a^ ± 0.079	386.2^c^ ± 14.34
T2	1.52^b^ ± 0.073	320.8^b^ ± 21.98

Different superscripts in 1 column indicate significant differences (p < 0.05); C(−): Chickens are given commercial feed only; C(+): Chickens given contaminated feed exposed to mycotoxin mix; T1, T2: Chickens were exposed to a mycotoxin mix with aflatoxin dose of 0.1 mg/kg feed + ochratoxin 0.1 mg/kg feed and mycotoxin binder with a dose for T1 of 1.1 g/kg feed while for T2 with a dose of 1.6 g/kg feed; Treatment for 8–35 days; Repetition=5 times. FCR=Feed conversion ratio

Together, these indicators offer a comprehensive evaluation of the physiological and productive responses of broilers to mycotoxin exposure and binder supplementation.

## DISCUSSION

### Effect of mycotoxicosis on broiler performance

Toxin binders are essential for chickens with mycotoxicosis caused by feed contaminated with mycotoxins. Chickens exposed to mycotoxicosis exhibit a decline in performance indices, including slaughter body weight, FCR, harvest age, and survival rate during rearing. Moreover, mycotoxicosis is associated with an increased FCR, reflecting reduced feed efficiency. This condition can occur due to a decrease in appetite, resulting in interference with immunity, specifically through the occurrence of an inflammatory process associated with mycotoxicosis infection.

### Residue reduction and tissue regeneration by toxin binders

Incorporating toxin binders into feed additives facilitates the elimination of ingested mycotoxins, thereby reducing residue levels in the meat and organs, such as the liver and intestines, of broiler chickens. Thus, residues from the mycotoxin mix in meat and organs such as the liver and intestines in the body of broiler chickens can be eliminated. In this study, the elimination of mixed mycotoxins, including AFB1 and OTA, was evaluated qualitatively and quantitatively using HPLC. subsequently, liver and intestinal tissue regeneration was assessed to assess the repair of mycotoxin-induced damage through regenerative processes.

### Broiler feed efficiency and production indicators

Broiler chickens, distinct from Leghorn chickens, are specialized for rapid growth and meat production. Broiler quality is primarily assessed using FCRs and performance indices [5, 9, 10]. The FCR was calculated by comparing total feed intake to body weight gain during the rearing period [5]. The FCR was calculated to determine the chicken’s ability to convert the feed it consumes into meat [10].

### Determinants of performance index in broiler production

The performance index is a critical determinant of the success rate of broiler farming operations [5, 10]. The performance index can be measured based on several parameters, such as ready-to-slaughter body weight, harvest age, FCR, and the total percentage of birds that survive during the rearing of broiler chickens [11]. The parameters influencing the performance index are largely dependent on the quality of feed consumed by broilers. High-quality feed is essential for the optimal growth and health of broiler chickens [5, 12].

### Importance of feed quality and storage conditions

Critical aspects of feed production include the selection of raw materials, processing methods, adherence to nutritional standards, proper formulation, mixing techniques, and contaminant control. Maintaining optimal temperature, humidity, cleanliness, and appropriate storage duration, along with microbial control, is vital for preserving feed quality [12].

### Mycotoxin contamination and fungal ecology in tropical climates

Molds that produce mycotoxins are major contributors to feed spoilage. The growth and development of mold are influenced by both climatic conditions and geographical location in a country. Indonesia’s high temperatures, rainfall, and humidity create favorable conditions for mold proliferation. Molds with high toxigenicity produce secondary metabolites that can form mycotoxins, which are hazardous to humans and animals, such as aflatoxin and ochratoxin. Mycotoxins are the result of secondary metabolites produced by certain molds, such as *Aspergillus flavus* and *Aspergillus parasiticus*, which produce aflatoxin. In addition, *Aspergillus ochraceus* produces ochratoxin [2, 4].

### Toxicological effects of aflatoxin and ochratoxin

Aflatoxins and ochratoxin present in raw or finished feed exert detrimental effects on livestock. Aflatoxins primarily damage the liver, alter gastrointestinal morphology and histology, impair nutrient digestibility, and reduce poultry productivity [13]. Similarly, OTA adversely affects the intestine, which is a highly sensitive target organ [14]. The effects in- clude weight loss due to decreased feed intake. Additionally, exposure to mycotoxins results in the suppression of immune function in poultry. Residues accumulated in the meat, liver, and intestines of poultry pose significant health risks to human consumers [15].

### Mycotoxin prevention and detoxification approaches

Prevention and detoxification of mycotoxins can be achieved through effective management during production, starting from the planting, harvesting, and storage of feed raw materials or finished feed. Additionally, the use of mycotoxin binders or mycotoxin binders found in feed additives can also be considered. Toxin binders, which are formulated as feed additives, are incorporated to mitigate mycotoxin contamination [16, 17]. The primary objectives of adding toxin binders are to reduce feed contamination, protect animal health, and minimize mycotoxin residues in animal-derived products [4].

### Stem cells as a biological repair mechanism

Endogenous stem cells are versatile cells capable of regenerating and differentiating into various cell types within specific tissues [6]. The differentiation of these cells is guided by induction agents that direct them into particular cell types [18, 19]. Types of endogenous stem cells, such as hematopoietic, mesenchymal, germline, and neural stem cells, support the maintenance of tissue function. When tissues are injured or homeostasis is disrupted, stem cells are activated to repair and regenerate tissues [6, 8]. They contribute to tissue repair through interactions with extracellular matrix, growth factors, the immune system, and cellular signals [6].

### Mechanisms of stem cell mobilization and homing

The activation of endogenous stem cells for tissue repair involves the mobilization, homing, and differentiation of these cells. This activation can be enhanced by natural or synthetic substances. Toxin binders, which contain natural components, may act as activators for endogenous stem cells, promoting repair and regeneration in tissues such as the heart, intestines, ovaries, and testes. The process involves mobilizing stem cells from their niche into the bloodstream, where they are transported to the damaged area, contributing to tissue regeneration [20].

Stem cell mobilization can occur through several mechanisms, including responses to inflammatory signals (e.g., nuclear factor kappa B, interleukin-1, tumor necrosis factor-alpha), immune responses, pharmacological agents, and specific blockers. This mobilization helps stem cells exit their niche and enter the bloodstream, enabling them to reach damaged tissues [21]. Studies have shown that natural ingredients can enhance stem cell mobilization and repair, as evidenced by improved liver tissue regeneration and the repair of intestinal and ovarian tissues [22, 23].

The homing of stem cells to damaged tissues involves the recruitment of signaling molecules from the microenvironment. This process can be local or systemic, with stem cells either migrating directly to the injury site or circulating through the body until they reach the target organ [24, 25]. Natural and synthetic agents can improve homing by enhancing the signaling pathways that guide stem cells to the damaged area, potentially offering therapeutic solutions for tissue regeneration [26]. This homing involves complex interactions with adhesion molecules and growth factors, ensuring that stem cells effectively reach and repair injured tissues [27–29].

### ADG

The findings revealed that the C+ group, which received feed contaminated with mycotoxins, exhibited the lowest daily weight gain compared with the control groups, C− and T1. Adding a mycotoxin binder at 1.1 and 1.6 g/kg of feed to groups T1 and T2 significantly improved body weight gain in broilers exposed to mycotoxins compared to group C+ (p < 0.05), though it did not show a significant difference when compared to the C− group (p > 0.05).

Body weight gain reflects the metabolic processes associated with broiler growth [5]. As shown in Table 5, the group exposed to mycotoxins without a mycotoxin binder exhibited the lowest daily body weight gain, which was significantly different from both the control group and the group receiving the binder. Aflatoxin contamination in feed adversely affects broiler weight gain because of reduced protein intake and disruption of protein metabolism [3, 30].

**Table 5:** Intraday precision of aflatoxin B1 dissolution in the mobile phase.

Sample	Area chromatogram	Std concentration (µg/mL)	Concentration (µg/mL)
C−	0.65674	0.0234	0.0022
C+	4.97095	0.0234	0.0179
T1	0.82066	0.0234	0.0029
T2	0.62031	0.0234	0.0023

Before they are metabolized, dietary proteins are absorbed by the microvilli in the digestive tract and then converted into amino acids for meat formation [31]. Direct exposure to mycotoxin-contaminated feed damages the gastrointestinal epithelium, potentially impairing nutrient abso-rption [32]. The epithelium helps maintain gastro-intestinal tissue balance by regenerating lost or damaged cells through a process known as apoptosis. Mycotoxins can disrupt this apoptosis mechanism by affecting cell proliferation.

The statistical analysis revealed a significant increase in daily weight gain in both the control group and those fed with an additional mycotoxin binder. The mycotoxin binder contains algae-derived nutrients, such as beta-carotene, which converts to vitamin A and supports cell proliferation in the digestive tract. Phytogenic compounds in the binder, including complex terpenoids, help reduce inflammation [33]. With vitamin A, these compounds contribute to maintaining gastrointestinal mucosal health and enhancing nutrient absorption, leading to healthier growth. In the digestive tract, mycotoxin binders act preventively by binding mycotoxins and facilitating their excretion through feces.

### FCR

The FCR measures the efficiency of converting feed to weight gain. A lower FCR indicates that broiler chickens are more effective at converting feed to meat [34]. In research analyzing the impact of mycotoxin binders on this ratio, the Duncan’s test revealed significant differences (p < 0.05).

As shown in Table 1, broiler chickens fed a mycotoxin-contaminated mix (AFB1 0.1 mg/kg feed + OTA 0.1 mg/kg feed) and treated with a mycotoxin binder at a dosage of 1.1 g/kg feed exhibited the best results, as evidenced by a lower FCR compared to other groups. The mycotoxin binder, which functions as a feed additive to enhance growth and feed efficiency, helped reduce the FCR, indicating improved feed efficiency.

The mycotoxin binder includes Hydrated Sodium Calcium Aluminosilicates and bentonite, which assist in binding aflatoxins in the digestive tract of broiler chickens. Bentonite, an aluminum silicate adsorbent, attaches to aflatoxins, rendering them non-toxic and facilitating their excretion through feces [35]. This binding action reduces the toxicity of mycotoxins in the digestive tract, enhances nutrient absorption, and improves feed efficiency.

Additionally, the binder contains other compo-nents such as BBSH 979 microorganisms, phytogenic ingredients, phycophytic substances, and a synergistic blend of minerals and biological elements. BBSH 979 microorganisms, including trichothecene-deactivating bacteria, help to convert mycotoxins into harmless metabolites. Phytogenic ingredients, a mixture of plant extracts, mitigate the adverse effects of mycotoxins and other toxic agents. Phycophytic substances from algae modulate the immune response and counteract the immunosuppressive effects of mycotoxins. The synergistic blend of minerals, processed into porous microballs, selectively adsorbs mycotoxins. Biological elements assist in the enzymatic decomposition and degradation of mycotoxins, such as trichothecenes and zearalenone, into non-toxic metabolites.

### Performance index

The performance index is one of the criteria used to evaluate the success and performance of broiler chickens over a certain period. A high-performance index value indicates that the performance of broiler chickens is improving and feed conversion is efficient [36]. The results of the study are in accordance with Table 1 in the group of broiler chickens that were fed feed contaminated with mycotoxin mix (AFB1 0.1 mg/kg feed + OTA 0.1 mg/kg feed) and given mycotoxin binder with a first dose of 1.1 g/kg feed, showing a result of 386. The performance index is categorized into five criteria, namely <300 less, 301–325 sufficient, 326–350 good, 351–400 very good, and >400 excellent [10].

The results from the treatment group of broiler chickens given feed contaminated with the first dose of the mycotoxin binder were categorized as excellent, with a value of around 351–400. The performance index can be increased by improving the DOC quality, maintenance, and feed management [37, 38]. The performance index was calculated based on harvest weight, mortality percentage, harvest age, and FCR [38, 39]. In this study, no deaths occurred in the broiler chickens. The harvest age used is when the broiler chicken is 35 days old.

The results of adding mycotoxin binder, included in the feed additive at a dose of 1.1 g/kg feed, yielded a higher performance index compared to other experimental groups. At this dose, broiler chickens could absorb the substances contained in the mycotoxin binder (feed additive) to the maximum extent. At higher doses, it is suspected that the mycotoxin binder not only absorbs the mycotoxin but also other nutrients, potentially wasting these essential nutrients.

The addition of a mycotoxin binder, which is included in the feed additive, is needed to help bind the mycotoxins in the feed. These binding agents can help bind mycotoxins, preventing them from being directly absorbed by the intestines. Mycotoxins that contaminate feed may reduce nutrient absorption. Mycotoxins damage the villi in the intestine, impairing nutrient absorption and preventing nutrients from flowing through the blood vessels, which affects the performance index in broiler chickens.

## CONCLUSION

This study demonstrated that dietary supple-mentation with a commercial toxin binder effectively mitigated the adverse effects of mixed mycotoxin contamination (AFB1 and OTA) in broiler chickens. HPLC analysis confirmed a significant reduction in AFB1 and OTA residues in broiler meat, particularly in the T2 group (1.6 g/kg), where AFB1 and OTA levels were reduced to 0.0023 μg/mL and 0.073 μg/mL, respectively. Furthermore, flow cytometric analysis indicated that the same dose significantly mobilized endogenous HSCs (CD34+, CD45+, CD105−), with an average expression of 35.62% ± 2.16, suggesting enhanced tissue regenerative capacity. The most optimal performance outcomes, including improved average daily growth (68.78 ± 4.78 g/day), the lowest FCR (1.38 ± 0.079), and the highest performance index (386.2 ± 14.34), were observed in the T1 group (1.1 g/kg binder).

These findings support the dual functionality of toxin binders – not only as detoxifying agents to ensure meat safety but also as potential enhancers of physiological resilience and growth performance in poultry. The use of 1.1 g/kg binder was sufficient to yield optimal production efficiency, while 1.6 g/kg facilitated superior detoxification and stem cell activation. Such applications could significantly benefit poultry producers in regions vulnerable to mycotoxin contamination.

The integration of residue quantification, stem cell biology, and performance metrics offers a novel and comprehensive evaluation of toxin binder efficacy. The use of validated HPLC methods and flow cytometry ensured methodological robustness and accurate detection of biomarkers.

The study did not include baseline mycotoxin screening of the commercial feed before artificial contamination. In addition, while stem cell induction was assessed, downstream tissue-specific regenerative effects (e.g., histological repair) were not examined.

Future studies should explore the long-term effects of toxin binder supplementation on tissue repair, immune modulation, and reproductive outcomes. Investigating the molecular mechanisms linking mycotoxin detoxification with stem cell activation may also provide mechanistic insights and inform precision nutrition strategies.

In conclusion, toxin binder supplementation, particularly at 1.1–1.6 g/kg, is a promising intervention to enhance food safety, protect broiler health, and improve production outcomes. These findings hold substantial relevance for poultry industries facing challenges from climate-driven increases in mycotoxin prevalence and underscore the broader potential of bioactive feed additives in regenerative animal production systems.

## AUTHORS’ CONTRIBUTIONS

ES: Coordinated the research, conceptualized and designed the study, and revised the manuscript; MWJ: Data collection and interpretation and drafted the manuscript. HP, TDL, SU, SD, RR, and ML: Contributed to the study design, data analysis, and critical review of the manuscript for important intellectual content. MT and GJ: Management of chicks, feed preparation, and edited the manuscript. All authors have read and approved the final version of the manuscript.
